# Striatal Lacunar Infarction in a Late Preterm Infant Born to a Mother with Active Peripartum SARS-CoV-2 Infection

**DOI:** 10.1155/2023/1611451

**Published:** 2023-09-28

**Authors:** Christoph Hochmayr, Marlene Hammerl, Ira Winkler, Gisela Schweigmann, Ursula Kiechl-Kohlendorfer, Elke Griesmaier, Anna Posod

**Affiliations:** ^1^Department of Pediatrics II (Neonatology), Medical University of Innsbruck, Innsbruck, Austria; ^2^Department of Radiology (Pediatric Radiology), Medical University of Innsbruck, Innsbruck, Austria

## Abstract

**Background:**

The current literature suggests that neonatal severe acute respiratory syndrome coronavirus 2 (SARS-CoV-2) infections generally have a mild course. Data on how in utero exposure to maternal infection affects neonatal health outcomes are limited, but there is evidence that neurological damage to the fetus and thromboembolic events may occur. *Case Presentation*. We describe the case of a late preterm infant, who presented with striatal lacunar infarction in the neonatal period, born to a mother with active peripartum SARS-CoV-2 infection. Diagnostic workup did not identify risk factors apart from the maternal SARS-CoV-2 infection. Repeated reverse transcription-polymerase chain reaction (RT-PCR) tests for SARS-CoV-2 using oropharyngeal swab specimens of the patient were negative. IgG, but not IgM antibodies against spike protein S1 receptor-binding domain (S1RBD) epitope were detectable in umbilical cord blood and neonatal serum collected at 48 hours of life. Anti-SARS-CoV-2 total antibody titers against nucleocapsid protein in umbilical cord blood were negative.

**Conclusions:**

Bearing in mind a possible association of in utero exposure to SARS-CoV-2 and neonatal thromboembolic events, neonatologists should be aware of these complications even in well-appearing preterm infants.

## 1. Background

With the rise of the COVID-19 pandemic, concerns grew about the impact of SARS-CoV-2 on particularly vulnerable populations such as newborns. Evidence suggests that neonatal SARS-CoV-2 infections are rare and generally have a mild course [1]. However, in utero exposure to a maternal SARS-CoV-2 infection may lead to a hyperinflammatory response and prothrombotic state in the fetus, potentially resulting in brain damage [2, 3]. In addition, inflammatory processes in the placenta can lead to thromboembolic events [4, 5]. In this report, we describe the unique case of a late preterm infant, who presented with unilateral striatal lacunar infarction in the neonatal period, born to a mother with active peripartum SARS-CoV-2 infection.

## 2. Case Presentation

The patient was born at 34 + 2 weeks of gestation with a birth weight of 2890 g to a 38-year-old third-gravid mother. The mother had a positive SARS-CoV-2 RT-PCR test on a nasopharyngeal swab five days prior to delivery and mild coronavirus disease 2019 (COVID-19) symptoms (headache, limb pain, sore throat, rhinitis, and shortness of breath); she was not vaccinated. Relevant medical history of the mother included gestational diabetes with very satisfactory dietary management; no relevant medication intake was reported.

**Figure 1:**
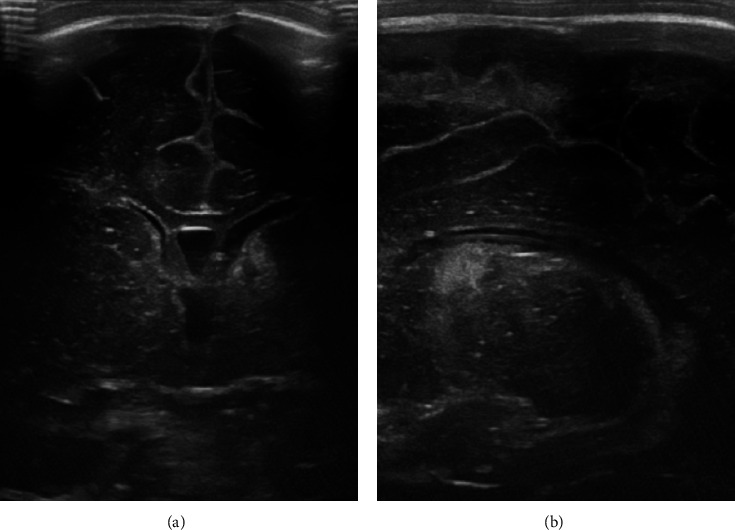
Cerebral ultrasound imaging. Ultrasound imaging shows demarcation of the increased echogenicity in the left subependymal area and basal ganglia: (a) coronal plane and (b) parasagittal plane.

The girl was delivered by secondary cesarean section due to unstoppable preterm labor and prior cesarean sections. The cesarean section was performed with an epidural block, and continuous monitoring of maternal vital signs and cardiotocography were normal throughout labor and delivery. Due to the uneventful initial course, there was no reason for performing placental histology, and the placenta was discarded after birth. Apgar scores were 7-8-9 at 1, 5, and 10 minutes, respectively, and umbilical cord arterial pH was 7.40 with a base excess of +0.9 mmol/L. In the delivery room, the baby was suctioned once because of airway obstruction due to mucus and insufficient breathing effort. She received five sustained inflations, followed by intermittent mask ventilation with a T-piece resuscitator (max. FiO_2_ 0.5) for 5 minutes. The heart rate increased adequately, and the girl subsequently showed sufficient breathing efforts and stable saturation measurements on room air, but displayed mild signs of respiratory distress. She was admitted to the Neonatal Intensive Care Unit and was put on continuous positive airway pressure support (FiO_2_ 0.21) for 10 hours. A chest radiograph showed mild diffuse granular opacification of both lungs consistent with the respiratory distress syndrome. Heart rate monitoring, pulse oximetry, and blood pressure measurements were normal throughout the observation period. A peripheral venous catheter was established and empiric antibiotic treatment was started on the first day of life. Comprehensive newborn examinations were normal. Empiric antibiotic treatment was discontinued after two days, as infection parameters were repeatedly negative. A blood culture and a culture of gastric aspirate returned negative. Complete blood count with differential results was repeatedly normal. Detailed routine laboratory results are provided in Table 1. Nasopharyngeal swabs taken on the first, fourth, and eighth day of life were negative for SARS-CoV-2. Routine cerebral ultrasonography on the fifth day of life showed increased echogenicity in the left subependymal area and basal ganglia; five days later, considerable demarcation occurred (as shown in Figure 1).

**Figure 2:**
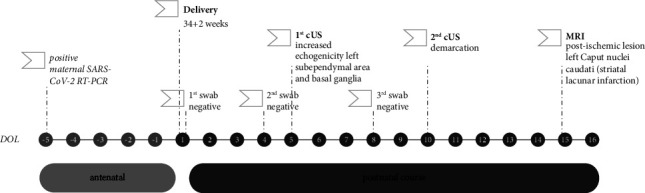
Timeline indicating relevant data from the episode of care. DOL: day of life; SARS-CoV-2: severe acute respiratory syndrome coronavirus 2; RT-PCR: reverse transcription-polymerase chain reaction; cUS: cerebral ultrasound; MRI: magnetic resonance imaging.

A timeline with relevant data from the episode of care is presented in Figure 2.

### 2.1. Diagnostic Assessment, Therapeutic Intervention, Follow-Up, and Outcomes

**Figure 3:**
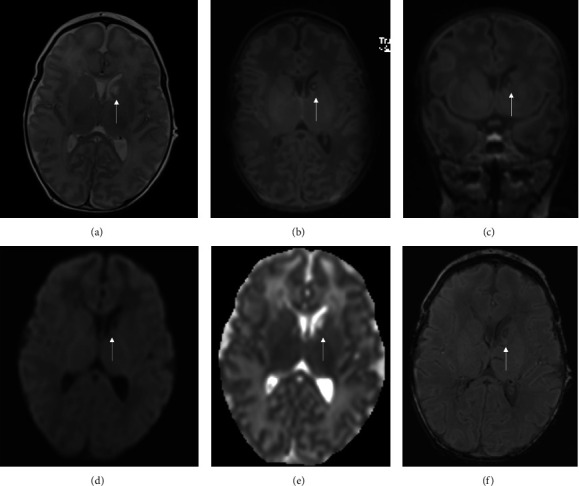
Magnetic resonance imaging (MRI) findings on the 15th day of life. MRI findings reveal a postischemic lesion in the left Caput nuclei caudati, consistent with striatal lacunar infarction with small central bleeding (white arrow). Axial T2 sequence shows white matter hyperintensity (a). Corresponding intensity on axial (b) and coronal (c) T1-weighted sequence. High-signal intensity at apparent diffusion coefficient (ADC) map (d) and low-signal intensity at diffusion-weighted imaging (e). Beginning of transformation at susceptibility-weighted imaging (SWI) (f).

At the age of two weeks, magnetic resonance imaging revealed a postischemic lesion in the left Caput nuclei caudati, consistent with striatal lacunar infarction (as shown in Figure 3). Time-of-flight magnetic resonance imaging angiography ruled out pathologies of the intracranial arteries. Treatment consisted of supportive care, as no benefit of an anticoagulant therapy was to be expected in a clinically and neurologically well-being of the patient with no evidence of a large vessel occlusion.

Serological testing of umbilical cord blood and neonatal serum collected at 48 hours of life showed anti-SARS-CoV-2 IgG antibody (S1RBD epitope) titers of 30.0 U/mL and 40.1 U/mL, respectively. Anti-SARS-CoV-2 IgM antibodies (S1RBD epitope) were negative in both sample types. Anti-SARS-CoV-2 total immunoglobulins against nucleocapsid protein in umbilical cord blood were negative in congruence with the very recent maternal infection. Pro- and anti-inflammatory cytokine levels in umbilical cord blood were as follows: tumor necrosis factor alpha 25.0 (<32.0) pg/mL, interleukin-6 6.7 (<10.1) pg/mL, interleukin-8 15.0 (<28.0) pg/mL, and interleukin-10 1.3 (<3.5) pg/mL. Further evaluation revealed no familial predisposition to thromboembolic events. Maternal coagulation studies (see additional Table 1) were normal and testing for lupus anticoagulant and anticardiolipin antibodies were negative. Routine serology screening for TORCH infections was also negative.

The remainder of the clinical course was uneventful. The newborn was fully breastfed for one month, and afterwards nutrition was switched to infant formula. Weight gain was appropriate at all times. The girl did not develop neurological signs or symptoms throughout the hospital stay and was discharged on the 16th day of life. The assessment of general movements at three months of corrected age showed age-appropriate movement patterns. The neurological assessment at a corrected age of 5 months revealed functional asymmetry of the right lower extremity and we initiated biweekly physical therapy. At a corrected age of 12 months, the girl showed normal neurological development.

## 3. Discussion and Conclusions

An increasing amount of the literature deals with the effects of SARS-CoV-2 on fetal and neonatal health. Neonatal infections generally have a benign course [1], but whether and how maternal SARS-CoV-2 infections during pregnancy affect neonatal outcomes is still incompletely understood. Prenatal SARS-CoV-2 exposure may cause a fetal and neonatal multisystem inflammatory response [6, 7]. Studies also suggest that in utero exposure to SARS-CoV-2 imprints the fetal immune system, leading to increased counts of natural killer cells, regulatory T-cells, and higher amounts of cytokines possibly causing alterations of fetal neurological development [2, 8, 9]. Furthermore, there has been a recent report on neonatal cerebral venous thrombosis, possibly owing to the prothrombotic state induced by maternal SARS-CoV-2 infection [3].

Arterial thromboembolic events such as ischemic stroke seem to be a rare complication in SARS-CoV-2-infected children, and even rarer in neonates. In an international survey, Beslow et al. report a single case of a 4-day-old neonate with a positive test result for SARS-CoV-2 and a thalamocapsular infarction [10]. We are the first to present a case of ischemic stroke in the neonatal period in the absence of an active SARS-CoV-2 infection or a multisystem inflammatory response in the neonate, but in association with maternal peripartum SARS-CoV-2 infection.

Neonatal cerebral infarction describes the occlusion of neonatal cerebral vessels due to arterial ischemic stroke (>90% of cases) or hemorrhagic infarction within the first 28 days of life [11]. Pathologic placental processes are the most probable etiology; it is hypothesized that the thrombotic emboli form in the fetal circulation of the placenta then pass the umbilical cord and cross the left heart via a patent foramen ovale or ductus arteriosus [5, 11]. Risk factors include primiparity, bacterial sepsis, amniotic inflammation, and fetal vascular malperfusion [5]. Examinations of placentas from SARS-CoV-2-infected mothers have shown inflammatory changes, fetal and maternal vascular malperfusion, as well as thrombotic lesions, underlining the procoagulant potential of SARS-CoV-2 and indicating a possible cause of neonatal ischemic stroke [4, 12].

In our patient, routine cerebral sonography incidentally revealed a lacunar infarction in the well-being of a late preterm infant. Diagnostic workup ruled out sepsis and inflammation; there was no indication of other infectious agents, as routine serological testing for TORCH during pregnancy was normal and repeated oropharyngeal swabs were negative for SARS-CoV-2. IgG antibodies against S1RBD were detectable in the umbilical cord and patient blood, consistent with placental transfer. Total antibodies against nucleocapsid protein were not detectable, which is in accordance with the recent maternal infection five days prior to delivery. Cytokine levels were within a normal range, making a hyperinflammatory state unlikely. The patient had no central venous catheters; no further thromboembolic events occurred, and there were no signs of an underlying prothrombotic disorder. Maternal diagnostic workup did not reveal additional risk factors for perinatal arterial ischemic stroke. Unfortunately, placental histology was not performed in our case, as the placenta was discarded after birth owing to the uneventful initial course, which is the major limitation in our line of argument. Nevertheless, bearing in mind the reported placental alterations observed in SARS-CoV-2-infected women and the pathophysiology of perinatal stroke, placental pathology, very probably linked to the maternal peripartum SARS-CoV-2 infection, remains the most likely etiology for the thromboembolic event observed in our case.

We cannot provide a direct link between the active maternal peripartum SARS-CoV-2 infection and the neonatal lacunar infarction in our patient. However, the observation that neonatal ischemic stroke may occur after in utero exposure to SARS-CoV-2 should sensitize neonatologists and specialists entrusted with the care of newborns for this complication. A causal relationship between maternal peripartum SARS-CoV-2 infections and thromboembolic events in the neonate remains to be proven, and it is necessary to collect more data in order to understand pathophysiological mechanisms of in utero exposure to SARS-CoV-2. Therefore, we suggest serological screening for SARS-CoV-2 in neonates who develop thromboembolic events, which cannot be explained by classical risk factors. We also recommend considering a low threshold for cerebral sonography in preterm neonates and term neonates with known risk factors for thromboembolic events born to mothers with active peripartum SARS-CoV-2 infection.

## Figures and Tables

**Table 1:** Routine laboratory results (reference ranges for each value are given in brackets).

Analysis of patient's blood	Day 1	Day 2	Day 16
C-reactive protein, mg/dL (0.0–0.5)	<0.06	<0.06	—
Interleukin-6, ng/L (0.0–100.0)	7.8	—	—
White blood cell count, ×10^9^/L (13.0–38.0)	15.1	10.1	11.6
Neutrophil count, ×10^9^/L (2.0–7.0)	3.6	5.1	1.8
Lymphocyte count, ×10^9^/L (3.38-21.6)	10.12	3.81	8.54
Segmented granulocytes, % (23.0–58.0)	24.0	50.7	15.9
Eosinophils, % (0.0–7.0)	1.0	0.7	4.3
Basophils, % (0.0–2.0)	0.0	0.0	0.0
Monocytes, % (8.0–13.0)	8.0	10.1	5.8
Lymphocytes, % (26.0–57.0)	67.0	37.8	73.9
Normoblasts, % (0.0-0.0)	4.5	1.7	—
Myelocytes, % (0.0-0.0)	—	0.7	—
Erythrocytes, ×10^12^/L (4.5–6.2)	3.8	3.34	3.28
Hemoglobin, g/L (140–230)	142	121	116
Hematocrit, L/L (0.45–0.62)	0.386	0.323	0.320
Mean corpuscular hemoglobin (MCH), pg (33.0–40.0)	37.4	36.2	35.4
Mean corpuscular volume (MCV), fL (91.0–102.0)	101.6	96.7	97.6
Mean corpuscular hemoglobin concentration (MCHC), g/L (300–370)	368	375	363
Red cell distributional width (RDW), % (11.5–14.4)	16.9	15.9	13.9
Platelet count, ×10^9^/L (213–515)	512	288	637
Immature platelets, % (0.8–6.3)	2.5	2.4	
Immature platelet count, ×10^9^/L (2.3–12.7)	12.8	6.9	
Fragmentocytes count,/HPF (0-1)	3–5	3–5	3–5
Reticulocytes, % (0–10)	—	62.2	—
Reticulocytes count, ×10^9^/L (0–60)	—	207.7	—
Alanine aminotransferase, U/L (4–49)	—	—	24
Aspartate aminotransferase, U/L (14–77)	—	—	8
Gamma-glutamyl transferase, U/L (8–178)	—	—	100
Alkaline phosphatase, U/L (83–248)	—	—	315
Total bilirubin, mg/dL (0.1–12.6)	2.0	8.6	9.6
Direct bilirubin, mg/dL (0.0–2.1)	—	—	0.63
Urea, mg/dL (9.0–41.0)	—	—	18.8
Creatinine, mg/dL (0.3–0.7)	—	—	0.34

## Data Availability

The data that support the findings of this study are available from the corresponding author upon reasonable request.

## Abbreviations

COVID-19: Coronavirus disease 2019
RT-PCR: Reverse transcription-polymerase chain reaction
SARS-CoV-2: Severe acute respiratory syndrome coronavirus 2
S1RBD: Spike protein S1 receptor-binding domain.

## Supplementary Materials

Supplementary Materials As additional materials, we offer the CARE checklist with indication of the lines where in the manuscript the respective items can be found. Additional table 1 displays prepartal coagulation studies of the mother.
