# Supplementary material for: Striatal Lacunar Infarction in a Late Preterm Infant Born to a Mother with Active Peripartum SARS-CoV-2 Infection
Source: Case Rep Pediatr. 2023 Sep 28;2023:1611451. doi: 10.1155/2023/1611451 (PMC10555493; doi:10.1155/2023/1611451)
Supplement: Supplementary Materials — As additional materials, we offer the CARE checklist with indication of the lines where in the manuscript the respective items can be found. Additional table 1 displays prepartal coagulation studies of the mother. [file 1611451.f1.zip › CARE-checklist-Hochmayr.pdf]

CARE case report guidelines

CARE Checklist of information to include when writing a case report

(cc) BY-NC-ND

Topic	Item	Checklist item description	Reported on Line
**Title**	**1**	The diagnosis or intervention of primary focus followed by the words "case report"	p1, paragraph 1
**Key Words**	**2**	2 to 5 key words that identify diagnoses or interventions in this case report, including "case report"	p 2, line 37
**Abstract (no references)**	**3a**	Introduction: What is unique about this case and what does it add to the scientific literature?	p2, Background, par 1
	**3b**	Main symptoms and/or important clinical findings	p2, Case present., par 2
	**3c**	The main diagnoses, therapeutic interventions, and outcomes	p2, Case present., par 2
	**3d**	Conclusion—What is the main "take-away" lesson(s) from this case?	p2, Conclusions, par 3
**Introduction**	**4**	One or two paragraphs summarizing why this case is unique (**may include references**)	p3, line 41-48
**Patient Information**	**5a**	De-identified patient specific information.	p3, line 50-51
	**5b**	Primary concerns and symptoms of the patient.	p3, line 56-67
	**5c**	Medical, family, and psycho-social history including relevant genetic information	p3, line 51-55
	**5d**	Relevant past interventions with outcomes	p3, line 60-67
**Clinical Findings**	**6**	Describe significant physical examination (PE) and important clinical findings.	p4, line 68-69, p8 117-118
**Timeline**	**7**	Historical and current information from this episode of care organized as a timeline	p6, line 85
**Diagnostic Assessment**	**8a**	Diagnostic testing (such as PE, laboratory testing, imaging, surveys).	p4, line 72-79
	**8b**	Diagnostic challenges (such as access to testing, financial, or cultural)	N/A
	**8c**	Diagnosis (including other diagnoses considered)	p4, line 76-95
	**8d**	Prognosis (such as staging in oncology) where applicable	p8, line 122
**Therapeutic Intervention**	**9a**	Types of therapeutic intervention (such as pharmacologic, surgical, preventive, self-care)	p7, line 95-97
	**9b**	Administration of therapeutic intervention (such as dosage, strength, duration)	p7, line 95-97
	**9c**	Changes in therapeutic intervention (with rationale)	N/A
**Follow-up and Outcomes**	**10a**	Clinician and patient-assessed outcomes (if available)	p8, line 117-118
	**10b**	Important follow-up diagnostic and other test results	p8, line 119-122
	**10c**	Intervention adherence and tolerability (How was this assessed?)	N/A
	**10d**	Adverse and unanticipated events	N/A
**Discussion**	**11a**	A scientific discussion of the strengths AND limitations associated with this case report	p10, line 160-167
	**11b**	Discussion of the relevant medical literature **with references**.	p9, line 124-150
	**11c**	The scientific rationale for any conclusions (including assessment of possible causes)	p10, line 163-167
	**11d**	The primary "take-away" lessons of this case report (without references) in a one paragraph conclusion	p10, line 168-179
**Patient Perspective**	**12**	The patient should share their perspective in one to two paragraphs on the treatment(s) they received	N/A
**Informed Consent**	**13**	Did the patient give informed consent? Please provide if requested	**Yes** ☒ **No** ☐
