# Supplementary material for: Striatal Lacunar Infarction in a Late Preterm Infant Born to a Mother with Active Peripartum SARS-CoV-2 Infection
Source: Case Rep Pediatr. 2023 Sep 28;2023:1611451. doi: 10.1155/2023/1611451 (PMC10555493; doi:10.1155/2023/1611451)
Supplement: Supplementary Materials — As additional materials, we offer the CARE checklist with indication of the lines where in the manuscript the respective items can be found. Additional table 1 displays prepartal coagulation studies of the mother. [file 1611451.f1.zip › Hindawi Case Reports in Pediatrics_Hochmayr_additional Table 1_Prepartal coagulation studies of the mother.docx]

| Prepartal coagulation studies of the mother |  |
| --- | --- |
| Prothrombin time (PT) | 116 (70 – 130) |
| International normalized ratio (INR) | 0.9 (0.0 – 6.0) |
| Activated thromboplastin time (APTT) | 23 (26 – 37) |
| Fibrinogen | 535 (210 – 400) |
| Antithrombin III | 112 (79 – 112) |
|  |  |
